# Supplementary material for: Effect of Cobalt Ferrite Nanoparticles in a Hydrophilic Shell on the Conductance of Bilayer Lipid Membrane
Source: Membranes (Basel). 2022 Nov 5;12(11):1106. doi: 10.3390/membranes12111106 (PMC9692745; doi:10.3390/membranes12111106)
Supplement: Supplementary file 1 [file membranes-12-01106-s001.zip › membranes-1973768-supplementary.pdf]

Materials and Methods

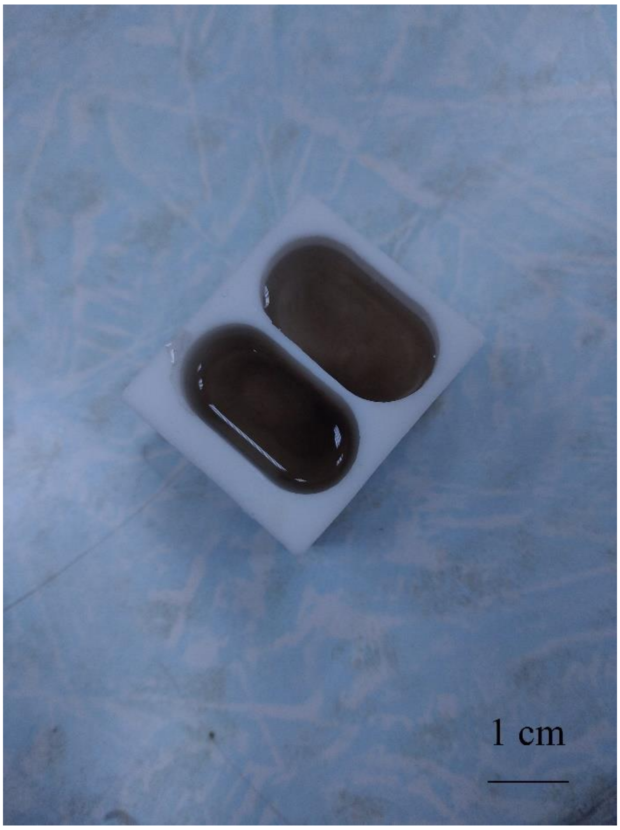

a)

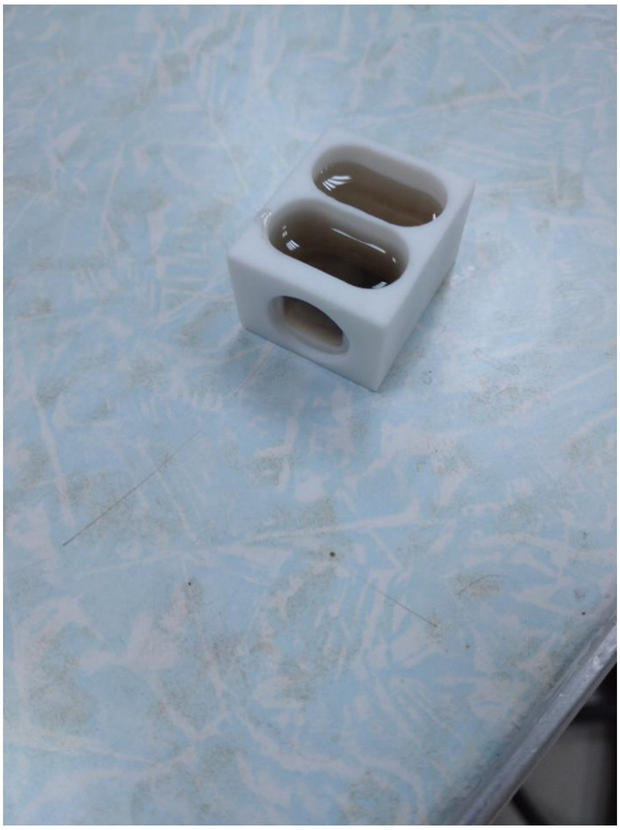

b)

Figure S1. MNP27 PEG is in Teflon cuvette. 100 μl MNP is added in *cis*-side of the cuvette and 60 μl MNP is added in *trans*- side of the cuvette.

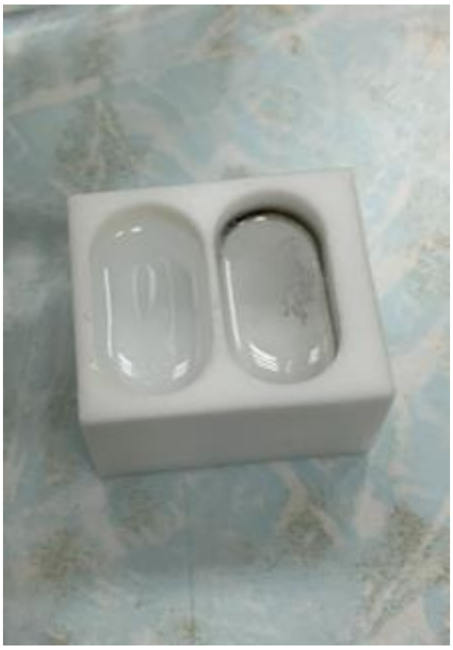

Figure S2. $CoFe_2O_4$ MNP dissolved in toluene (without hydrophilic coating) is in Teflon cuvette.

Results

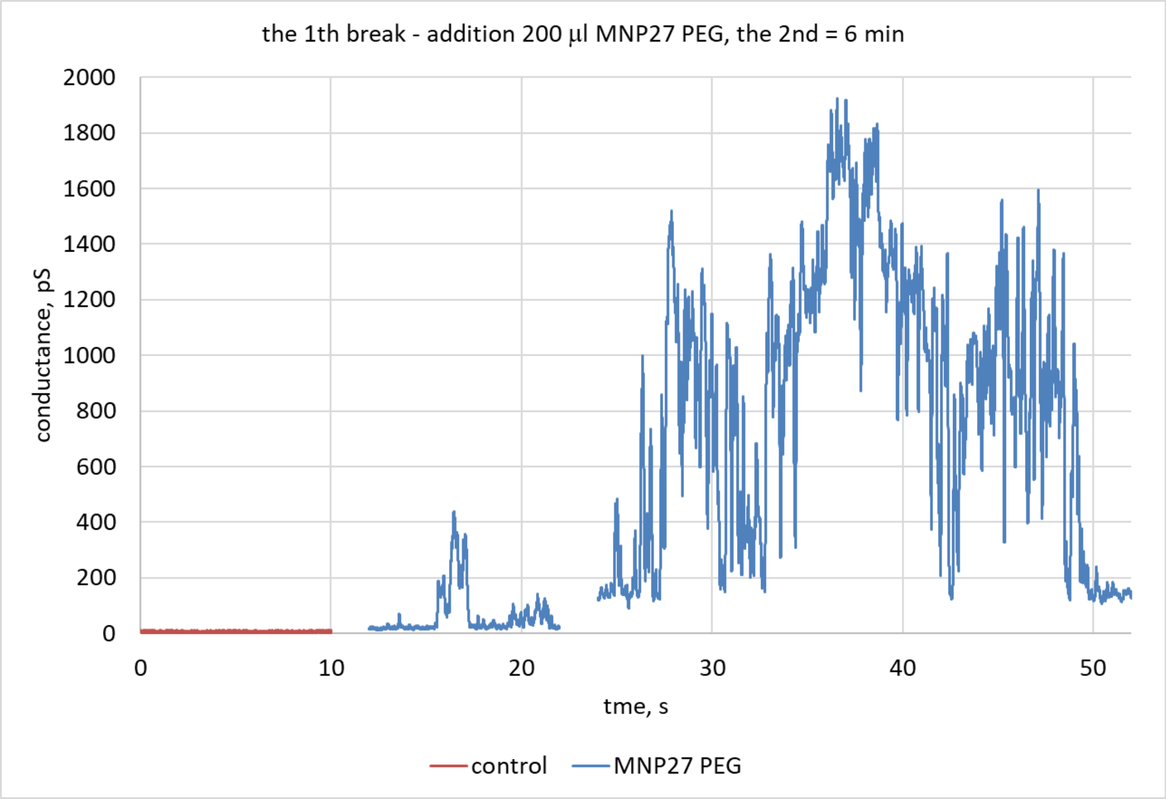

a)

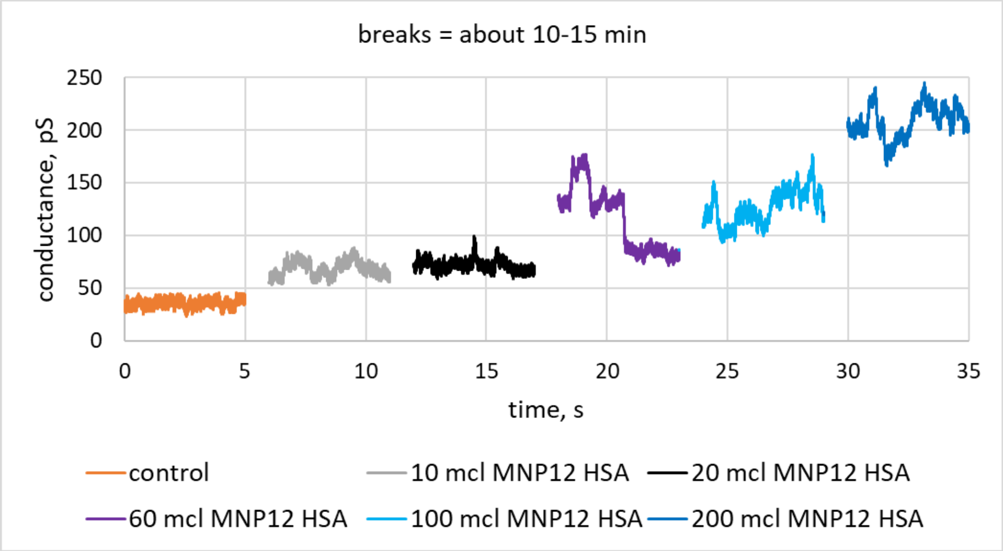
breaks = about 10-15 min
conductance, pS
250
200
150
100
50
0
0
5
10
15
20
25
30
35
time, s
control
10 mcl MNP12 HSA
20 mcl MNP12 HSA
60 mcl MNP12 HSA
100 mcl MNP12 HSA
200 mcl MNP12 HSA

b)

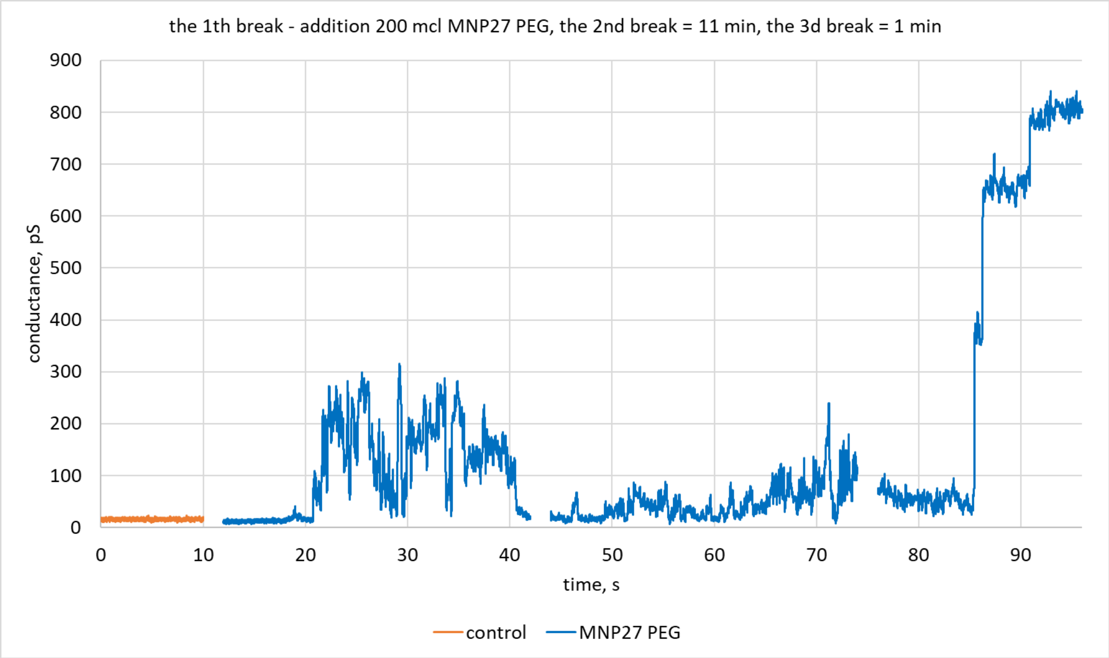
the 1th break - addition 200 mcl MNP27 PEG, the 2nd break = 11 min, the 3d break = 1 min
conductance, pS
900
800
700
600
500
400
300
200
100
0
0
10
20
30
40
50
60
70
80
90
time, s
control
MNP27 PEG

c)

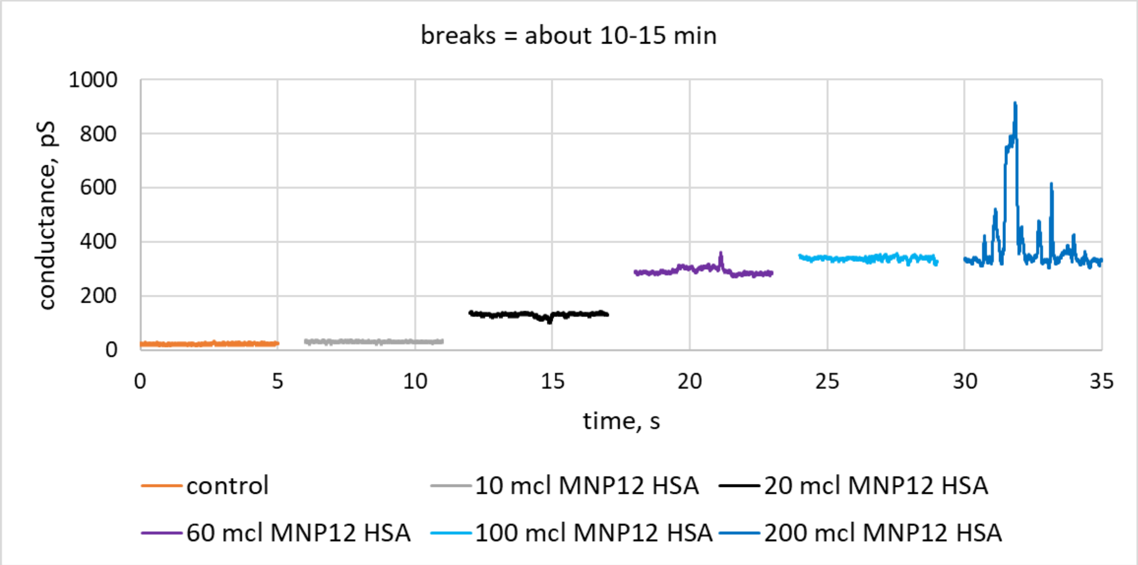
breaks = about 10-15 min
conductance, pS
1000
800
600
400
200
0
0
5
10
15
20
25
30
35
time, s
control
10 mcl MNP12 HSA
20 mcl MNP12 HSA
60 mcl MNP12 HSA
100 mcl MNP12 HSA
200 mcl MNP12 HSA

d)

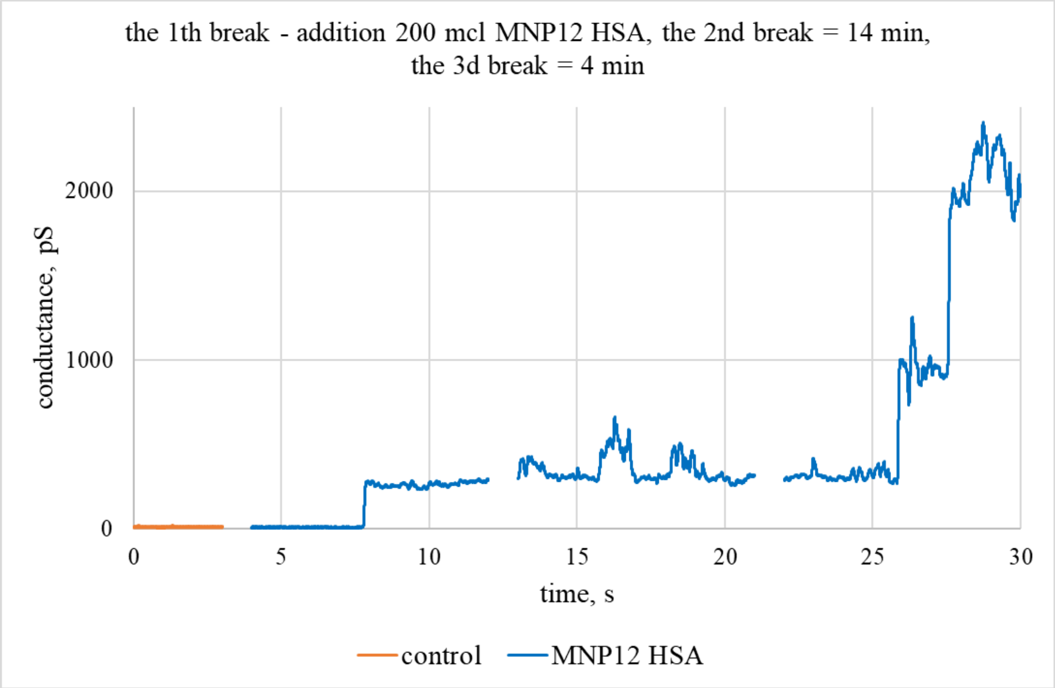
the 1th break - addition 200 mcl MNP12 HSA, the 2nd break = 14 min, the 3d break = 4 min
conductance, pS
time, s
control
MNP12 HSA
e)

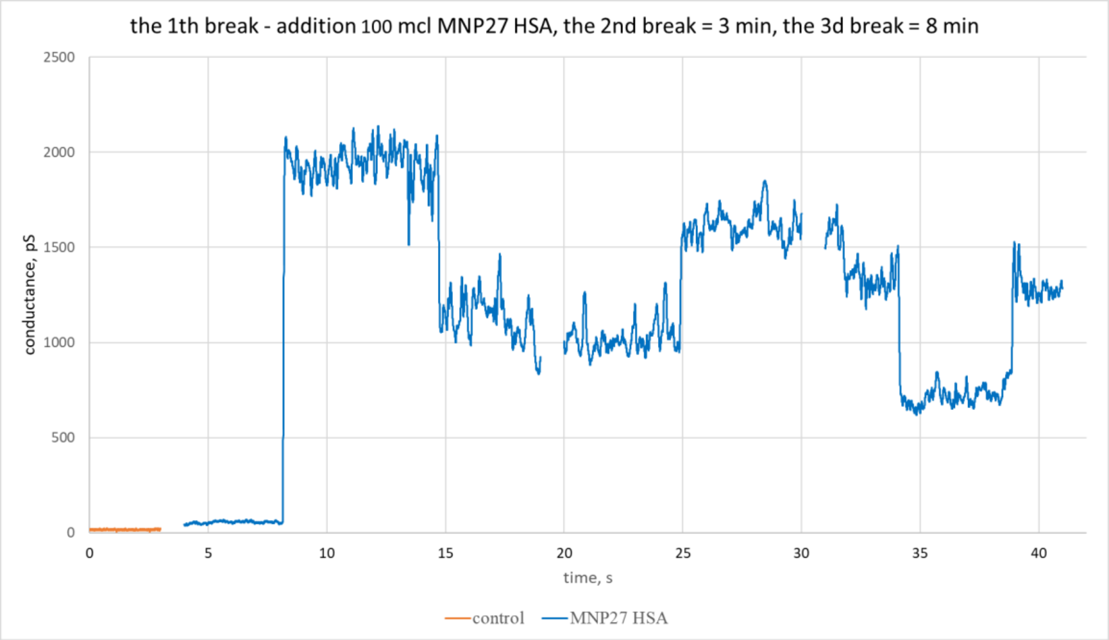
the 1th break - addition 100 mcl MNP27 HSA, the 2nd break = 3 min, the 3d break = 8 min
conductance, pS
time, s
control
MNP27 HSA
g)

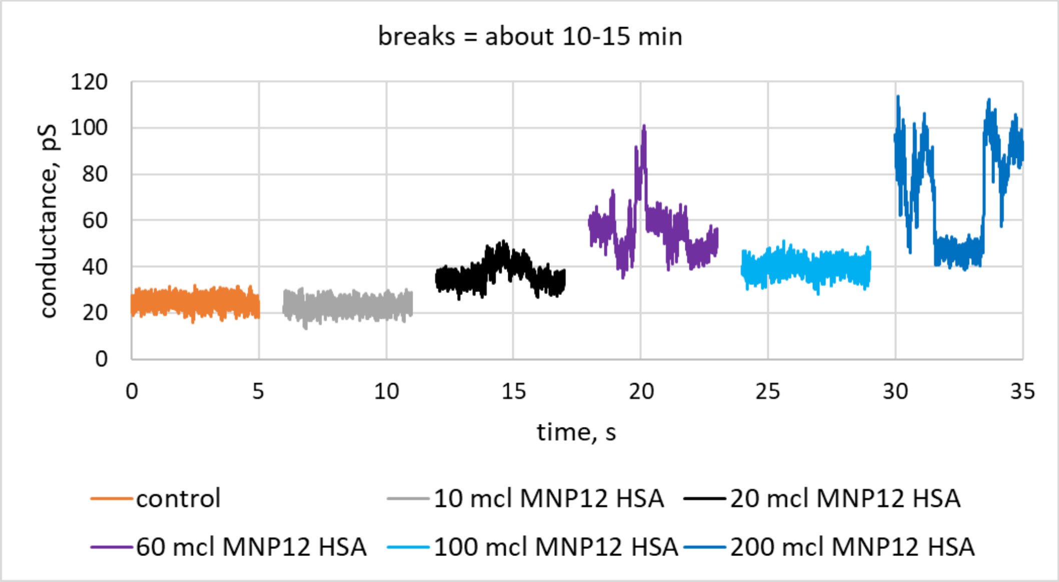

h)

Figure S3. Examples of conductance traces for different membranes.
